# “CRISPR for Disabilities: How to Self-Regulate” or Something?

**DOI:** 10.1007/s11673-021-10162-8

**Published:** 2022-04-01

**Authors:** Amanda Courtright-Lim

**Affiliations:** 1 Cardiff University, Cardiff, Wales CF10 3AT UK; 2 Translational Genomic Research Institute, 445 N. 5th Street, Phoenix, AZ 85004 USA

**Keywords:** Bioethics, Self-regulation, Biotechnology, Gene editing, Disability, Basic research

## Abstract

The development of the CRISPR gene editing technique has been hyped as a technique that could fundamentally change scientific research and its clinical application. Unrecognized is the fact that it joins other technologies that have tried and failed under the same discourse of scientific hype. These technologies, like gene therapy and stem cell research, have moved quickly passed basic research into clinical application with dire consequences. Before hastily moving to clinical applications, it is necessary to consider basic research and determine how CRISPR/Cas systems should be applied. In the case of single gene diseases, that application is expected to have positive impacts, but as we shift to more complex diseases, the impact could be unintentionally negative. In the context of common disabilities, the level of genetic complexity may render this technology useless but potentially toxic, aggravating a social discourse that devalues those with disabilities. This paper intends to define the issues related to disability that are associated with using the CRIPSR/Cas system in basic research. It also aims to provide a decision tree to help determine whether the technology should be utilized or if alternative approaches beyond scientific research could lead to a better use of limited funding resources.

## Introduction

The hot topic in genetics over the past few years has been gene editing, particularly usage of the CRISPR/Cas system (Gyngell, Douglas, and Savulescu 2017; Reardon 2015; Cyranoski 2015). The discourse around this technology has been heated at times, including fears among the general public of this leading to “designer babies” and scientists in disagreement about how to move forward with a strategy for real-world applications (Hampton 2016; Reardon 2015). Much of the conversation has become fixed on the possibility of editing the germline, the point in a cell’s life where any change in genetics will be passed on to subsequent generations. At the moment, there appears to be agreement among both scientists and the public that germline genetic editing should be avoided (Blendon, Gorski, and Bensen 2016). Scientists are apprehensive of using the germline editing technology in humans for two reasons. The first is that our understanding of the human genome is still limited, and with the initial mapping of the human genome completed only a decade ago, it is still too early to begin editing the genome (Lander 2015). The second reason is that germline editing beyond a few specific cases would be considered to be enhancement, which could make the public resistant to using gene editing in general, even in somatic cells, which will not pass on the genetic change (Lanphier et al. 2015).

Agreement around the potential dangers of germline editing has led to regulations being put in place. These include the revision to the pan-European regulation on clinical trials, which has prohibited gene therapy clinical trials that modify a subject’s germline, as well as the 2015 International Summit on Human Gene Editing meeting in Washington D.C. finding that clinical research was premature and additional research was needed ahead of germline editing as a therapy (Isasi, Kleiderman, and Knoppers 2016; National Academies of Sciences, Engineering, and Medicine 2015). Although clinical applications may be on hold with germline cells, with the exception of a single case in China, there have been fewer limitations placed on basic research on germline gene editing (Wang et al. 2018). As basic research in this area continues, it has the potential to lead to a clinical application as the regulatory landscape changes. In this case, it is not only important to consider clinical applications of this technology, once it has been developed but the developments in basic research itself that shape what these clinical applications could be.

These considerations lead to the objective of this paper, which is to evaluate the ethical dimensions of moving from basic research on the CRISPR/Cas system to a clinical application and providing a method of self-regulation, via a decision tree, to assess when this technology should be developed and applied. After a brief review of gene editing in Section 2, Section 3 and Section 4 of this paper explore the complexity of genetic risk factors in the context of complex disease and common disabilities to demonstrate the potential negative impact of using the CRISPR system. In Section 5, a proposition will be made to use a decision tree for self-regulation of germline gene editing research that will be explored using disability-based examples to demonstrate its application. Bringing together all the aspects of this paper, the aim is to reconsider the application of CRISPR on germline gene editing research.

## Gene Editing

Before exploring the topics of disease and disability, it is necessary to first look at gene editing and determine why the newest advances have the scientific community so excited. The idea of editing our genetic material is not a new idea. The development of recombinant DNA technology in the 1970s was the first time that scientists were able to alter the sequence of DNA bases and edit the genome (Hsu et al. 2014). The caveat with this technology is that targeting the genome through recombinant methods is time-consuming, expensive, and can be unreliable. The recent discovery of the CRISPR/Cas system looks to alter the landscape of editing the human genome as it is fast, cheap, and looks to be potentially reliable.

The discovery of the Clustered Regularly Interspaced Short Palindromic Repeats (CRISPR) system dates back to the late 1980s, but a full understanding of the technology would not be made until decades later. As scientists explored different organisms through the sequencing of their genomes, researchers came across the CRISPR/Cas system in prokaryotic cells, where it is used as an RNA-mediated adaptive defence system (Charpentier 2015). This naturally occurring system is important to molecular biologists because the Cas9 gene, found in one of the systems variations, can target and modify DNA. Like the tools of a Swiss Army knife, this ability to target and modify DNA forms the basis of the current use of the CRISPR/Cas system. In simplistic terms, the CRISPR element acts as a compass to guide the system to a certain location and the scissors, Cas9 proteins, are able to cut and add/remove the target sequence. This, in combination with mechanisms that already exist on the host cell, allow for it to be used quickly and at a much lower cost than previous methods of genome editing (Charpentier 2015). The CRISPR/Cas system developments have stimulated the hype around gene editing technology and experimentation around human germlines.

In 2015, a team of Chinese scientists led by Junjiu Huang shocked the scientific world by announcing that they had attempted to edit the human germline using CRISPR/Cas9. This was the first time the CRISPR/Cas system was being used in this way and it raised many concerns regarding the safety of this type of research. One of the key conversations at this time was about the resulting off-target effects that were observed in human tri-pronuclear zygote germline cells that were used (Liang et al. 2015). Heated conversations arose, not just among ethicists but between the scientists themselves, about the unknown risks that the CRISPR/Cas system could cause to the germline. A number of pivotal meetings took place to discuss the future of this gene editing technology. Everyone was in agreement that this technology could have a great impact on treating disease, but the risks, such as off-target effects and misuse of the technology, led to the introduction of limits to the funding of human germline editing in the clinical space. This did not limit basic research though, and in 2016, Shoukhrat Mitalipov’s team from Oregon Health Sciences University were able to successfully edit the human germline (Ma et al. 2017). This experiment was pivotal for two reasons: one was that they were using CRISPR to target a disease-causing gene and the second was they had no notable off-target effects. This not only validated the power of the technology but also endorsed the argument it was the next tool that would change molecular medicine.

This key development in the CRISPR story adds to an already divided conversation among both scientists and the general public. Even with its pivotal advances, this technology may not live up to the positive hype promoted by enthusiasts and could in fact end up having a negative impact. To begin assessing the impact, it is necessary to look at the spaces that CRISPR could affect, starting with consideration of how disease and disability are framed.

## Disease

Disease is defined as a disorder of either structure or function in a living organism that results in a particular set of symptoms (Oxford University Press 2021). Disease, in this context, refers to a condition that is not caused by physical injury. The public understanding of genetics has advanced in the decade after the completion of the human genome and, with it, so too has our understanding of disease. Both society and science have moved past the model of believing that all diseases are caused by a single genetic marker to a model where they are caused by a complex system. The latter embraces the fact that environment, heritable change, and even chance act as disease factors beyond genes. Moving towards this model of understanding alters the considerations that should be in play with basic research. With single gene disorders, of which there are more than six thousand known, the rate of occurrence is relatively low, making them rare diseases (Loi 2012). Cystic fibrosis is estimated to affect 1 in 2,500 births in the United Kingdom annually, and over seventy thousand total cases worldwide (Cystic Fibrosis Trust 2021: Cystic Fibrosis Foundation 2021). Although that seems like a large number and not a very rare event, we can better understand its stature when compared to a common disease like Alzheimer’s Disease, which the World Health Organization estimates to affect around 35.6 million individuals worldwide as of 2010 (Duthey 2013). The contrast in numbers is evident when looking at rare diseases as opposed to common diseases, but it is the underlying genetics that sets some common conditions apart as complex diseases.

Imagine walking into a dark room. The common reaction is to turn on the lights, except that in this room, there is a myriad of switches both nearby and on the other side of the room. In order to determine which switch controls the lights, it is necessary to turn on every switch encountered until the correct one is found. Now imagine that several of the switches do not directly control the light, but instead control the quality of the light—the colour and the brightness. Unless you try every combination, you cannot predict the brightness or colour of light from any switch that is flipped. The idea of genetic risk factors (GRFs) can be thought of like this dark room scenario, where attempting to alter its current state can have unforeseeable, mixed consequences. For example, a change to a GRF may turn off the risk of disease in one set of cells but now turns on the risk of disease in another set of cells (Dupras and Ravitsky 2016). A relevant case study of this phenomenon is the way that genes affect both cancer and neurodegeneration. One recurring theme in neurodegenerative disease research is that if a gene is upregulated in cancer, which propagates cell growth, then it must be downregulated in neurodegeneration, which causes the death of cells (Klus et al. 2015). Even though this example is somewhat reductionist, it has been found to be true (Ukraintseva et al. 2016; Plun-Favreau et al. 2010). Research indicates that the many molecular mechanisms associated with each disease have an overlap, meaning that many of the same pathways may be affected, simply in a different manner (Morris, Veeriah, and Chan 2010). In the cases of both Alzheimer’s Disease (AD) and Parkinson’s Disease (PD), they have a much lower representation of cancer survivors with the opposite being seen in relation to a cancer diagnosis among those with AD and PD (Houck, Seddighi, and Driver 2018).

Accordingly, a key concern of GRFs is that they may have adaptive features that have not been adequately understood at this time. A review paper on GRFs in human longevity showed that many GRFs could act as either risk alleles for specific diseases or pro-longevity variants, depending on the situation (Ukraintseva et al. 2016). Therefore, “risk” may not be the appropriate word or frame of mind to be utilized if GRFs have positive and negative trade-offs for their presence in a genome and the more appropriate term may be genetic “variant.” This indicates that it might be time to modify how we think about the human genome and the markers found coded in our DNA associated with disease.

Because manipulating genetic variants is likely to have many unpredictable and negative downstream effects, basic research into complex disease is therefore far from guaranteed to have a clinical pay-off. Limiting basic research in this space would face significant resistance from both the scientific community and the public. Nonetheless, there is a space where a push to consider both the positive and negative aspects of genetic variation—and consequently change how certain conditions are viewed—and that is the space of disability. In the context of disability, societal factors have a large impact on how conditions are perceived, even when genetic variants are present, raising concerns with the purpose of basic research in this area. To understand this fully, the next step in this paper is to consider the genetics associated with disabilities and current societal perspectives.

## Disability

The World Health Organization’s International Classification of Functioning, Disability and Health (ICF) defines disability as multi-dimesional, which considers aspects such as impairment, activity limitation, and participation restrictions in life situations (Kazou 2017; Patel and Brown 2017). This ICF looks to go beyond the concept of a condition and instead of focusing simply on the body’s function it includes the individual’s lived experience thereby allowing for interventions targeted at both the individual and their environment (Üstün et al. 2003). This breadth in what might be considered a disability is due, in part, to the vast variation found among disabilities. As is the case with rare but familiar diseases, there are well-known disabilities caused by genetic changes, such as Down Syndrome or Fragile X, but there are also conditions caused by genetic variation. In the context of the issues examined in this paper, it is important to keep the term “disability” broad so as to include less severe disabilities that are not typically discussed but nevertheless are covered by disability legislation in countries such as the United States and United Kingdom.

The group of disabilities considered here is Specific Learning Disabilities (SLDs), which describes conditions that do not affect intellectual ability, but instead creates a challenge to learning by traditional methods. SLDs were selected as the focal point not because of their potential for gene editing, but because there is limited scientific discussion about them. In addition, the exploration of such disabilities is enriched by decades of discourses from other disability studies, including those focused on changes in how disabilities are viewed. As SLDs have received less attention in scientific discourses than other conditions, such as Cystic Fibrosis, Spinal Muscular Atrophy (SMA), deafness, and Fragile X, this may provide a better chance of having genuine discussions about SLDs that are not dominated by predetermined views that a more commonly considered condition presents. This opens opportunities for diverse discussions focused more on the topic of disability rather than the current medical means for addressing it, a common theme in more commonly discussed disabilities. To get a better understanding of these issues, it is important to unpack SLDs in more detail.

SLDs impact a person’s education and typically have a limited impact on daily life. For the purposes of this discussion, this paper will focus on the SLD of dyslexia. The estimated rate for dyslexia is between 5 and 12 percent of the population, making it the most common neurodevelopmental disorder (Schumacher et al. 2007). There have been known genetic links to the condition for decades, with the most recent research pinpointing potential causes. For example, a number of genes have been shown to be associated with neuronal development; many having links to a defect occurring during the development of an individual’s brain in utero. However, Dorothy Bishop, a leading researcher on dyslexia, highlights in her work that genetics is not a final indication of the phenotype (Bishop 2015). Much of her early work on families showed a link between genetics and dyslexia, but in her studies of twins, not all sets of monozygotic twins were both affected with dyslexia (Bishop 2015). In addition, a recent publication reviewing the neuronal migration model of dyslexia questioned its accuracy and suggested that many early genetic markers have not replicated from previous cell culture work into an in vivo model (Guidi et al. 2018). This points towards a more complex model of genetics, as is seen with more common and complex diseases, in which alteration in genes related to dyslexia play out like genetic variants, presenting positive and negative changes.

Taking the disease comparison further, there are indicators, like symptoms, that can indicate the presence of an SLD. In the case of dyslexia, some key symptoms are being late to talk and slow to learn new words and, significantly, a delay in the ability to read (Mayo Clinic 2017). Even with these negative indicators, current discourse around dyslexia highlights the positive indicators that come with this disability. Research has shown that those affected with dyslexia have better visual-spatial ability, a type of thinking that aids in creative work (Kapoula et al. 2016). Whether this is a result of adapting to a brain that does not function in a traditional way, or a genetic change that reduces the capacity for one trait only to boost it for another, it is an interesting correlation to be explored. Chriss Gyngell and Thomas Gourglas highlight this connection when discussing liberal eugenics and ask us to consider how we look at the genetics of disabilities that affect cognition—not as a difficulty in learning but as a different way of thinking (Gyngell and Douglas 2015).

This way of thinking differently is not a new concept and can be found in the discourse from the field of disability studies looking at different models for understanding disabilities. One such model is the social model of disability, which argues that a disability is not caused by the actual impairment^1^ but is a result of social structures and attitudes towards the impairment (Oliver 2013: Oliver 1996).^2^ The social model is not the only model of disability, and the arguments in this paper do not rest on accepting it as such, but aspects of the social model are clearly applicable to the range of common disabilities discussed here. This notion extends to the idea of “neurodiversity,” which is the argument that disabilities that appear to have an effect on how the brain functions—like SLDs, Attention Deficit Disorder (ADD), and autism among others—are natural genetic variations that may, in fact, be adaptive (Griffin and Pollak 2009). For example, many people are advocating for value to be placed on the strength a person with dyslexia has, such as novel thinking and excellent problem-solving skills, instead of focusing on the reading and spelling deficits that individuals with dyslexia often have (Armstrong 2015; Garner 2021). Promotion of this perspective of neurological difference is occurs in the broader community, for example in public talks presented by the TED organization and through advocacy by famous individuals, like British businessman Richard Branson. Many studies around SLDs have found that social barriers, such as education systems that restrict unconventional thinking, are what shape the lived experiences of and the social and medical responses to conditions such as SLDs (Bacon and Bennett 2013; Denhart 2008; Griffiths 2011). Like some other diagnoses and categorizations, the history of the SLDs also highlights the significance of the social context. SLDs came into existence after The Children with Specific Learning Disabilities Act of 1969 (U.S.) was instigated to get pupils access to additional educational support (Backer 2002). To summarize, the complexity of an impairment goes beyond the genetics into the social context around disabilities. While this may not be the case for all impairments, what this indicates is that context will dictate how we value conditions and, depending on the context, it will shape the views on gene editing for disabilities.

If context is key when discussing these common disabilities, then different mentalities will influence the use of technologies used to treat them. Despite the social movement toward positively reshaping how disabilities like SLDs are seen, there is a strong possibility that basic research could be misused or prematurely applied in a clinical application, negatively affecting the favourable discourse toward the societal acceptance of these disabilities. This makes it necessary to explore attitudes and approaches to disabilities in the context of basic research, even before clinical applications become possible.

## Self-Regulation

Thus far, this paper has focused on more complex genetic conditions and has not addressed those conditions that have a definitive genetic diagnosis. This is not because the ethical questions in that latter space are not interesting or important but because it is generally agreed that definitive genetic conditions will be able to benefit from the clinical application of germline gene editing when it is ready (Blendon, Gorski, and Bensen 2016). The aim of going from basic research to clinical application is already in mind for single gene diseases and disabilities. In the case of complex diseases and disabilities, that trajectory for basic research to clinical application is less straightforward. Trying to decide the scope and funding of basic research exploring the possibility of gene editing of the germline for these complex diseases and common disabilities needs to be critically evaluated.

For decades, the idea of regulating scientific technology and placing limitations on research has placed bioethicists and scientists (among other stakeholders) in contention with each other. This usually means that an idea of placing a moratorium on a technology is often at odds with much of the scientific community. In the case of CRISPR, discussion of a freeze on germline gene editing came up at the International Summit on Human Gene Editing meeting in Washington D.C. in 2015, where many scientists supported enforcement of a moratorium on germline gene editing. The moratorium conversation resurfaced at the 2018 meeting, with news that Chinese scientist He Jiankui had gene edited embryos, leading to the birth of twin girls. Even with this ethically problematic incident taking place, no moratorium was put in place, in part because many scientists feared it would limit scientific research and potential progress (Hayden 2016). This resistance to regulation raises concerns about what basic research is being conducted using CRISPR gene editing and about a need for some level of regulation. In the current climate around CRISPR gene editing technology, a move toward self-regulation may be the only immediate way forward.

The aim with self-regulation is not to circumvent or replace traditional regulatory frameworks but to provide a tool for use while other regulatory methods are explored and applied. Traditional regulations, like direct government regulations, can be slow to establish or be delayed, as has been the case with the germline gene editing moratorium. The goal of self-regulation is to avoid this by placing relevant resources in the hands of the researchers and encouraging a deeper consideration about the implications of their research. This is especially so when researchers are cognizant of the ethical considerations around their research (Resnik and Elliot 2016). While this may not remove all misuses of research or a technology, it does strive for a cultural shift where a more complex ethical consideration is made ahead of a basic research study. This paper presents a tool in the form of a decision tree to assist with a self-regulation process for CRISPR basic research.

**Fig. 1:**
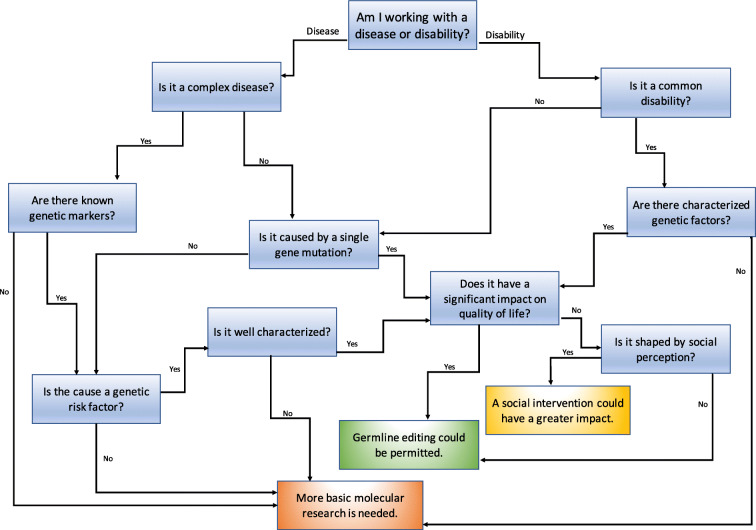
A decision tree to assist with selfregulation of CRISPR basic research

Any unnecessary complexity in a regulation tool risks alienating individuals from engaging with the process. When asking individuals to self-regulate, a tool must be designed in a way that it is accessible and encourages engagement (Gunningham and Rees 1997). A decision tree is a visually engaging tool that gives the user a straightforward understanding of the regulation process, including the factors to take into account and the potential outcomes. The decision tree (Fig. 1) provided in this paper has been designed with accessibility in mind. Each portion of the tree has been kept simple and how different aspects of consideration pass through the tree is clearly demonstrated. The clear-cut endpoints provide three options when evaluating gene editing in both diseases and disabilities: requiring more basic molecular research; a social intervention having a greater impact; and germline editing could be permitted. The endpoint “more basic molecular research is needed” identifies areas of research where the genetics is unclear and more research is needed before considering any form of gene editing. The second conclusion, “a social intervention having a greater impact,” is aimed at helping to identify conditions that, although genetically characterized, could be addressed over time through social intervention. The final endpoint “where germline editing could be permitted” is there to determine the few spaces associated with both disease and disability that would benefit from germline editing with well-characterized molecular mechanisms and that are not predominately shaped by societal views.

The way of working through the decision tree follows the same idea of user-friendliness. It was designed for basic researchers by a basic researcher, keeping in mind the variability that exists across the field. It is evident that there is no clear-cut line that separates disease and disability. To get a level of consistency, the decision tree was created to end up at the same three points no matter which path is chosen. If two different classifications of disease or disability are used for a condition, they will still converge in the same place. This is, in part, due to the interconnectivity designed into the decision tree. In addition, the terminology used within the tree is in line with the existing common and well-known terms in research, allowing researchers to work through the decision tree with ease. The overarching aim of this decision tree is to aid considerations of how to move forward on the topic of germline editing using the CRISPR/Cas system. Before applying it to that topic, it is useful to consider cases of misuse in order to understand how to apply the statements built into the tree. The potential for misuse of the CRISPR technology is one of the key aspects discussed in relation to its application. When researchers publish their experiments in journal articles they report their methods to enable others to replicate the work. The potential misappropriation of this basic research into clinical applications is illustrated in the case of biotechnology company 23andMe. In 2013, 23andMe was ordered by the U.S. Food and Drug Administration (FDA) to halt marketing of its direct-to-consumer genetic testing, which included promotion of health-related genetic tests (Annas and Elias 2014). One of the key questions about the 23andMe product focused on the validity of the markers that were being tested (Baudhuim 2014). When the FDA allowed the company to begin testing again two years later, it was under the stipulation that it could only test for markers validated by research, meaning many of the markers only found in single studies had to be removed from its testing product (Boddy 2017). This case demonstrates an instance of a clinical application with limited research evidence. Using the decision tree proposed in this paper, the details of this case would lead to a decision that “more molecular research is needed,” which matches up with the FDA’s ruling.

Another example helpful to exploring and evaluating the potential misuse of basic research relates to stem cell therapies. Recently, the FDA has been shutting down clinics that specialize in unapproved stem cell therapy. Prior to the hype of CRISPR, some successful stem cell experiments on humans resulted in certain clinics touting stem cell therapy as a clinical technique. However many clinics offered untested methods and many cases have been found where patients experienced serious adverse effects. One famous case emerged from a stem cell therapy clinic in Florida, where three women paid to take part in what they mistakenly thought was clinical research, and their sight was lost due to complications with the untested therapy (Kuriyan 2017). If we look to the decision tree in the context of the FDA regulating stem cell clinics and examine the option “germline editing could be permitted,” a focus must be placed on the word “could.” In this case, even if stem cell therapy could have an impact, an untested method would not be permitted without strong basic research demonstrating its safety. The decision tree aligns with researchers in the stem cell field, such as Dr. Paul Knoepfler, who advocated against these unapproved stem cell therapy clinics (Knoepfler 2016). Tools like the decision tree can act as a framework within which voices critical of technology uses can be made audible, thereby helping decision-making around application of technologies, such as germline editing. To explore this on a broader scale, it is time to step away from past examples and explore the decision tree in the present, in the context of disability.

Based on this paper’s earlier assessment of dyslexia and the neurodiversity discourse, the decision tree points to “social intervention being a better route” than germline editing using CRISPR for common disabilities like dyslexia. To test the decision tree, it is useful to explore a more complex SLD, auditory processing disorder (APD). This is a disorder that results in the brain and the ear processing sound at different speeds (Bamiou, Musiek, and Luxon 2001). For individuals with APD, a loud classroom can be a challenging environment in which to learn. These difficulties extend beyond the classroom as people with APD can have a hard time in social settings, where picking up tones and varying sounds are necessary. Many of the features seen in APD, such as the hearing impact and the social impact, reflect those experienced in other disabilities like deafness and autism spectrum disorder (ASD). In the case of deafness, many people do not regard themselves as disabled because their loss of hearing gives them a cultural identity (Hintermair and Albertini 2005). This same discourse has led to the idea that deafness has been over-medicalized, and the field of medicine is making a negative judgment on the quality of life associated with deafness (Johnston 2015). When considering the decision tree and reaching the section that asks, “Does it have a significant impact on quality of life?”, context is key. For those who regard deafness as an impairment that negatively impacts quality of life, then basic research to understand it is worthy, which leaves gene editing as a possible option. Similarly, APD could also be considered by some in a medicalized context and gene editing determined as the goal. What this fails to recognize is the social model of disability and discourses indicating that disability is shaped by more than just genetics. This is recognized in the decision tree via the consideration of “Is it shaped by social perception?”, which opens the door to alternative approaches. The decision tree, therefore, draws attention to the influence of social perspectives on how disability is seen and that this needs to be considered in the basic research space.

The aim of the decision tree is that it be used by not only researchers but by funding agencies, institutional review boards, and ethics committees to determine the best use of resources. The gene editing technology CRISPR may be inexpensive but the tools used in conjunction with it, like next-generation sequencing, are still very costly. Therefore, in the space of disabilities where the genetics are still complex, putting resources into basic research that uses gene editing may not lead to a scientific impact and the money may be better used elsewhere. Approaches to SLDs, like APD and dyslexia, are still more based in educational rather than medicalized spaces. At the same time, education for people with disabilities remains an underfunded area in many Western countries. For example, the underfunding of the U.S. *Individuals with Disabilities Education Act* (IDEA) means States are expected to cover the missing resources and public schools are facing a funding crisis (Moor 2005). During 2018 alone, there were six state-wide walkouts by teachers demanding more funding in schools, many of which shut down schools and whole districts for extended lengths of times (Turner, Lombardo, and Logan 2018). This lack of funding limits the resources a school has to test for SLDs. An untold number of individuals go undiagnosed, leaving them to struggle in the classroom and not receive the tools that would put a value on their strengths. This is just the tip of the iceberg when it comes to the social considerations for SLDs and indicates that using a decision tree to look beyond genetics is necessary. That said, each disability will be different and looking outside genetics may not always be the result of the decision tree.

Another important factor is that disabilities are variable. Impairments that have the feature of being on a spectrum means the phenotype varies from individual to individual. In the case of many of the SLDs, this notion implies certain individuals may be affected more than others. If we take this variation into consideration, the resulting endpoints may not be the same when using the decision tree. This is evident in the case of ASD, which is also an SLD. Those with ASD can be severely affected by communication and/or intellectual impairments or on the other end of the spectrum they can fall into a category once known as Asperger’s in which they have difficulty with social cues but are often unaffected otherwise (Salyakina et al. 2010; Lintas and Persico 2009). Depending on which end of the spectrum an individual falls, the outcome of the decision tree may be different. There may be arguments for a need for germline editing in disabilities with more severe impacts. This is not a drawback but instead an indication of how the decision tree is shaped to look not solely at genetics, quality of life, or social aspects, but to consider them all together when attempting to reach a decision. In the case of ASD, the decision tree may never make it past the point that considers determination of genetic characterization, as ASD is a heterogeneous disorder, meaning the same condition is associated with different genes and genetic markers (Jiao et al. 2012; Freitag 2007). That would result in ASD falling into the endpoint that “more basic molecular research is needed,” which is built in to the tree to continue to allow research to take place with impairments that could lead to a treatment or intervention for those with that condition, if that is what the community desires.

The scientific community has become so caught up in the idea of curing and eliminating all disease and disability that it lost sight of whether that is truly what is needed. As a scientist, bioethicist, and an individual with a disability, I find myself questioning the direction science is heading in in that regard and I am not alone. As has been presented here, there are voices questioning our current direction with the use of the CRISPR technology. Placing our hope for answers on a given technology, and more broadly the discipline of science, discounts the fact that the answer may be elsewhere. If we shift from a focus on genetics and how we can manipulate it and look more broadly at the context in which genetics sits—a human—it will be possible to shift value onto actual persons and not solely their genetics and the negative perceptions of their impact. Moving toward using tools like CRISPR adds to the discourse that genetics is a key factor in determining what is of value in being human. Using this decision tree allows for the social context to come into consideration and move toward a social shift in how we see common disabilities over a genetically based one. As this frame of mind shifts, it can then open up the conversation into complex disease and, as a collective society, we can begin to decide if germline editing is the path to be chartered or if there is a safer path that we cannot yet see beyond the hype around CRISPR.

## Conclusion

Gene editing of the human germline has moved from fiction to reality. With it comes a necessity for some level of regulation so that there is no misuse of this technology. This is necessary because, when exploring disease and disability, it becomes apparent that the genetics is complex and that social views influence negative associations with some conditions and impairments. Furthermore, evaluating the advancements in gene editing technology enables us to scrutinise science at work, including the possible missteps and past misuses, which is vital to knowing what precautions to take in advancing gene editing technology. Just because we can now gene edit the germline does not mean we should. The decision tree presented in this paper helps demonstrate that there is more at play than just genetics in complex disease and common disability, upon which basic research using germline editing with CRISPR/Cas system will not have an impact. It might be time to step back from the hype of the latest scientific technology and remember that our genetics may not be the aspect of us that needs changing.

## Footnotes

1 Impairment representing the functional effects that are causing concern.

2 Other models of disability include the moral and/or religious model, medical model, the identity model, the human rights model, the cultural model, the economic model, the charity model, and the limits model.
